# The burden of antibiotic resistance of the main microorganisms causing infections in humans – review of the literature

**DOI:** 10.25122/jml-2023-0404

**Published:** 2024-03

**Authors:** Alexandru-Paul Baciu, Carmen Baciu, Ginel Baciu, Gabriela Gurau

**Affiliations:** 1 MedLife Hyperclinic Nicolae Balcescu, Galati, Romania; 2 Sf. Ioan Emergency Clinical Hospital for Children, Galati, Romania; 3 Faculty of Medicine and Pharmacy, Dunarea de Jos University, Galati, Romania

**Keywords:** antibiotic resistance, antibiotics, methicillin-resistant *Staphylococcus aureus*, beta-lactamase, carbapenems, vancomycin, AOM, acute otitis media, CDC, Centers for Disease Control and Prevention, cIAI, complicated intra-abdominal infection, CRE, carbapenem-resistant Enterobacterales, cUTI, complicated urinary tract infection, ESBL, extended-spectrum beta-lactamase, Hib, *Haemophilus influenzae* type b, LVRE, linezolid/vancomycin -resistant enterococci, MBC, minimum bactericidal concentration, MBL, metallo-beta-lactamases, MDR, multidrug-resistant, MIC, minimum inhibitor concentration, MRSA, methicillin-resistant *Staphylococcus aureus*, PBP, penicillin-binding protein, SCC*mec* staphylococcal chromosomal cassette *mec*, VRE, vancomycin-resistant enterococci, XDR, extensively drug-resistant

## Abstract

One of the biggest threats to human well-being and public health is antibiotic resistance. If allowed to spread unchecked, it might become a major health risk and trigger another pandemic. This proves the need to develop antibiotic resistance-related global health solutions that take into consideration microdata from various global locations. Establishing positive social norms, guiding individual and group behavioral habits that support global human health, and ultimately raising public awareness of the need for such action could all have a positive impact. Antibiotic resistance is not just a growing clinical concern but also complicates therapy, making adherence to current guidelines for managing antibiotic resistance extremely difficult. Numerous genetic components have been connected to the development of resistance; some of these components have intricate paths of transfer between microorganisms. Beyond this, the subject of antibiotic resistance is becoming increasingly significant in medical microbiology as new mechanisms underpinning its development are identified. In addition to genetic factors, behaviors such as misdiagnosis, exposure to broad-spectrum antibiotics, and delayed diagnosis contribute to the development of resistance. However, advancements in bioinformatics and DNA sequencing technology have completely transformed the diagnostic sector, enabling real-time identification of the components and causes of antibiotic resistance. This information is crucial for developing effective control and prevention strategies to counter the threat.

## INTRODUCTION

According to the World Health Organization (WHO), antimicrobial resistance is a “serious threat (that) is no longer a prediction for the future, it is happening right now in every region of the world and has the potential to affect anyone, of any age, in any country” [1]. Antibiotics are essential to contemporary medicine, being one of the most important factors in the reduction of infant mortality. Furthermore, they are essential for a wide range of medical procedures, from surgery to chemotherapy, and the treatment of secondary infections and of cancers of infectious origin. However, as multidrug-resistant bacteria become more prevalent worldwide, the threat of incurable diseases is becoming increasingly palpable. A 2022 report by the WHO stated that the world will run out of antibiotics because existing drugs have been developed by modifying existing classes and have been shown to have short cycles of impact [2].

According to a 2019 study, mortality caused by bacterial resistance to antibiotics exceeded 1.2 million over the 1-year study period, surpassing the number of deaths caused by human immunodeficiency virus (HIV) infection and malaria [3]. The same study concluded that over 1.27 million people could have been saved if antibiotic-resistant infections had been replaced by bacterial infections sensitive to common antibiotics [3]. Although the COVID-19 pandemic has captured the attention of the medical world in the last 3 years, antibiotic resistance remains an urgent problem that could lead to the appearance of much more aggressive, even lethal, pathogens in the near future [1].

It was found that mortality caused by increased antibiotic resistance has a higher rate in underdeveloped and developing countries, but remain a priority in developed countries as well [1]. Therefore, a global understanding of this problem is necessary. Deaths resulting from thoraco-abdominal and systemic infections represent 79% of all deaths caused by antibiotic resistance.

One of the most concerning aspects of antibiotic resistance is the occurrence of recurrent infections or secondary infections with saprophytic microorganisms in patients with underlying diseases or limited mobility, which leads in many cases to treatment failure [4,5].

The pathogens most frequently linked to antibiotic resistance-related mortality are *Acinetobacter baumannii, Pseudomonas aeruginosa (P. aeruginosa), Klebsiella pneumoniae (K. pneumoniae), Staphylococcus aureus (S. aureus),* and *Escherichia coli (E. coli)*. Furthermore, methicillin-resistant *S. aureus* (MRSA), vancomycin-intermediate S. aureus, and vancomycin-resistant S. aureus were incriminated as a major cause of mortality in a study that examined 88 pathogen–antibiotic combinations [1]. The same study showed that the resistance of bacteria to the first line of antibiotic treatment (used mainly empirically) is responsible for more than 70% of deaths. These antibiotics include fluoroquinolones and beta-lactamases, for example carbapenems, cephalosporins, penicillins, and ciprofloxacin [1].

In response to recent studies, the WHO listed the issue of antibiotic resistance as one of the top ten worldwide dangers to public health, highlighting that the primary reason of the increasing prevalence in antibiotic resistance is improperly delivered therapies (wrong dosages, overuse of antibiotics) [3]. The WHO also cautions that in certain parts of the world, inadequate sanitation and a shortage of drinking water can lead to the spread of bacteria, particularly those that are already resistant to available treatments [3].

## ETIOLOGICAL AGENTS OF IMPORTANCE IN THE ERA OF ANTIBIOTIC RESISTANCE

Among Gram-positive pathogens, a global pandemic of resistance of *S. aureus* and *Enterococcus* species currently represent the most significant threat [6,7]. According to studies conducted in the United States, MRSA is responsible for the deaths of more Americans each year than HIV/AIDS, Parkinson’s disease, emphysema, and homicide combined [8,9]. In addition, vancomycin-resistant enterococci (VRE) and a growing number of additional pathogens develop resistance to many antibiotics commonly used in medical practice [8]. The global spread of drug resistance of the pathogens most often involved in respiratory infections, *Streptococcus pneumoniae* (*S. pneumoniae*) and *Mycobacterium tuberculosis* (*M. tuberculosis*), has been classified as an epidemic [7].

Gram-negative pathogens represent a particularly alarming problem because they become resistant to almost all available antibiotic options, creating situations reminiscent of the pre-antibiotic era [6–8]. The appearance of Gram-negative multidrug-resistant (MDR) bacilli has affected the entire medical practice [8]. The most severe Gram-negative infections occur in hospital settings and are most commonly caused by Enterobacterales (especially *K. pneumoniae*), *P. aeruginosa*, and *Acinetobacter* [6]. MDR Gram-negative pathogens are also becoming more prevalent in the community and include *E. coli* and *Neisseria gonorrhoeae* (*N. gonorrhoeae*), which produce extended-spectrum beta-lactamases (ESBLs) [7]. These microorganisms are of significant clinical importance in hospitals because infected patients often require care in the intensive care unit (ICU) and are at high risk of morbidity and mortality.

### Streptococcus pneumoniae

*S. pneumoniae* remains the leading pathogen of acute bacterial pneumonia according to US statistics, although after the neonatal period, ~70% of cases of acute pneumonia are caused by viruses with respiratory tropism [10]. *S. pneumoniae* has the potential to produce severe and occasionally fatal infections, and is a major cause of bacterial pneumonia and meningitis, as well as blood, ear, and sinus infections [6,9]. Most cases of antibiotic resistance and deaths in infections with *S. pneumoniae* occur among adults aged 50 years or older, with the highest rates among those aged 65 years or older [6]. Data from vaccine trials for the heptavalent pneumococcal vaccine indicated that a third of radiologically confirmed cases of pneumonia were caused by *S. pneumoniae* [11]. However, a study by the Centers for Disease Control and Prevention (CDC), which investigated pathogens involved in radiologically confirmed pneumonias in hospitalized patients in three US hospitals, found a bacterial pathogen in only 15% of the children, and *S. pneumoniae* was the second most frequent cause of infection, the first being *Mycoplasma pneumoniae* [10].

Complicated pneumonia cases (necrotizing pneumonia, pleural empyema, lung abscess) represent a problem of worldwide importance, even after the introduction of the heptavalent vaccine [12]. Pleural empyema is most commonly determined by serotypes 1, 3, 7A, 18, and 19A, whereas forms with necrosis by serotype 3 of *S. pneumoniae* [12,13]. The rate of these complications has decreased in countries where the 13-valent pneumococcal vaccine was introduced into the vaccination schedule, although serotype 3 continues to be an important etiological agent of pneumonia with pleural empyema [14,15].

Historically, *S. pneumoniae* has been sensitive to penicillins, cephalosporins, macrolides, clindamycin, vancomycin, and trimethoprim-sulfamethoxazole. However, in the late 1990s, and then later in 2002 and 2008, strains of *S. pneumoniae* with a minimum inhibitory concentration (MIC) of >8 µg/ml appeared, changing the definition of pneumococcal sensitivity to penicillins and cephalosporins, from susceptible to intermediate and even resistant [16,17]. Resistance to penicillins correlates directly with resistance to broad-spectrum cephalosporins. After the introduction of the heptavalent vaccine, strains with increased resistance to macrolides were isolated (serotype 19A); the introduction of the 13-valent vaccine was followed by a decrease in pneumococcal macrolide resistance [18,19]. Linezolid and daptomycin have shown both in laboratory tests and in real-world settings to be effective against *S. pneumoniae*. From 1997 to 2016, 11 strains of invasive *S. pneumoniae* resistant to linezolid were isolated in the United States [20]. Very rarely, resistance to carbapenems has also been observed [21]. In 30% of severe infection with *S. pneumoniae*, the bacteria are completely resistant to one or more clinically relevant antibiotics [6].

After the introduction of the anti-pneumococcal vaccine into the mandatory regimen, a decrease in pneumococcal strains resistant to penicillins and, implicitly, third generation cephalosporins was observed [20,22], caused by a reduction in both pneumococcal colonization and antibiotic use.

Before the use of the heptavalent vaccine, risk factors associated with *S. pneumoniae* antibiotic resistance included being of white race, being a nasopharyngeal carrier or having a pneumococcal infection before the age of 5 years, recent antibiotic treatment, and residence in a community with high antibiotic consumption [23–25]. After the introduction of the heptavalent vaccine, antibiotic resistance to serotypes that were not covered by this vaccine increased [26]. The widespread use of the 13-valent pneumococcal vaccine was associated with an increase in nasopharyngeal carriers of non-vaccine strains, but the proportion of resistant strains isolated from the nasopharynx decreased in most studies [27,28], except for one, which highlighted that after an initial decline in resistance, there was a rebound in resistance, especially of serotype 35B [29].

### Streptococcus pyogenes

*Streptococcus pyogenes* (*S. pyogenes*) is the main bacterial pathogen that affects the pediatric age groups, especially young children and adolescents, and is associated with a range of diseases. It is estimated that there are ~600 million cases of acute *S. pyogenes* pharyngitis and ~700 million cases of *S. pyogenes* pyoderma cases worldwide [30]. Although the conditions generated by *S. pyogenes* are mostly benign, there is a risk of nonsuppurative sequelae such as acute articular rheumatism or acute poststreptococcal glomerulonephritis. Although *S. pyogenes* is still sensitive to penicillins and other antibiotics, it represents a public health problem because of the large number of annual cases, the complications that can occur, and the difficulty of diagnosis. In addition, *S. pyogenes* is an important cause of morbidity and mortality in developing countries, with >500,000 deaths related to acute respiratory failure and invasive infections [30].

In humans, the first step in the pathogenesis of diseases caused by *S. pyogenes* is represented by the colonization of the upper respiratory tract or the skin. The formation of the biofilm facilitates the persistence of the infection [31]. Both M protein and fibronectin contribute to the endocytosis of *S. pyogenes* into respiratory epithelial cells. This intracellular invasion of *S. pyogenes* has been postulated to be responsible for repeated infections after correctly administered antibiotic therapy, and repeated antibiotic courses might not lead to eradication but to the selection of more invasive strains [32].

*S. pyogenes* is susceptible to beta-lactam agents, but very rarely, strains have been isolated with a mutation of the penicillin-binding protein that confers moderate resistance, needing a higher MIC. Nevertheless, *S. pyogenes* remains susceptible to penicillins [33]. A 2019 study reported that a mutation in the penicillin-binding protein (PBP), *pbp2x*, resulted in enhanced resistance to ampicillin and amoxicillin in two patients with significant invasive infections with *S. pyogenes* subtype *emm43.4*. However, the study found no proof that this mutation is widely distributed [33].

In light of the above, the antibiotic of choice for *S. pyogenes* is penicillin, although amoxicillin and ampicillin are also widely used because of better acceptability by children and parents [34]. Given the resistance of *S. pyogenes* to macrolides, these are reserved for patients who are allergic to beta-lactam antibiotics. Sulfonamides and tetracycline are not effective and should not be used to treat strep throat.

There is evidence that cephalosporins are superior to penicillins and that they may reduce the number of chronic carriers after the end of treatment, but higher costs and the potential risk of developing resistance mean that cephalosporins are used as second-line therapy for patients who are sensitive to beta lactam antibiotics. Erythromycin is an alternative antibiotic for the treatment of certain *S. pyogenes* infections [34].

Some patients do not respond to treatment, which can be classified as bacteriological or clinical failure. When a patient shows symptoms despite a correctly administered treatment, retesting for *S. pyogenes* is necessary. If the culture is positive, then the treatment with beta-lactam antibiotics is resumed. If the culture is negative then the symptoms have another etiology. Bacteriological failure is classified as true or false. In the case of true bacteriological failure, the *emm* type of *S. pyogenes* persists despite correctly conducted treatment. There are several potential causes in the absence of penicillin resistance, such as tolerance to penicillin (the discrepancy between the concentration of penicillin required to inhibit/destroy the bacteria), the presence of pharyngeal flora that increases the colonization and growth of *S. pyogenes* or one that produces beta-lactamases that inactivate penicillin, or the internalization of *S. pyogenes* by the host cells, which protects it from the antibiotic [35,36].

### Staphylococcus aureus

One common bacterial pathogen that causes a wide range of clinical symptoms is *S. aureus*. Both community-acquired and hospital-acquired infections are common, and treatment remains difficult to manage owing to the emergence of multidrug-resistant strains such as MRSA [37,38].

MRSA strains carry a *mec* gene on the bacterial chromosome, which is a component of the larger Staphylococcal cassette chromosome *mec* (SCC *mec*) region, more specifically *mecA*, conferring resistance to several antibiotics depending on the type of SCC *mec* [37]. Penicillin-binding protein 2a (PBP-2a) is a protein that is encoded by the *mec* gene. The synthesis of peptidoglycan in the bacterial cell wall is catalyzed by PBP-2a even in the presence of several antibiotics because it has a lesser affinity than other PBPs for binding to beta-lactams and other penicillin-derived antibiotics. Because of this, MRSA strains are resistant to a wide range of antibiotics, and *S. aureus* strains that produce PBP-2a can proliferate in the presence of numerous drugs. Hence, MRSA strains are frequently resistant to methicillin, nafcillin, oxacillin, and cephalosporins [37,38].

MRSA was first discovered five decades ago [39]. Since then, MRSA infections have spread worldwide, occurring with high incidence in many countries in Europe, America, and Asia [7]. MRSA infections can be very severe and are one of the most common infections caused by antibiotic-resistant bacteria [6]. MRSA is resistant to penicillin-like beta-lactam antibiotics [40]. However, a number of antibiotics still retain activity against MRSA, including glycopeptides (e.g., vancomycin and teicoplanin), linezolid, tigecycline, daptomycin, and even some newer beta-lactams such as ceftaroline and ceftobiprole [7]. Nevertheless, MRSA has demonstrated exceptional adaptability in its development and dissemination within healthcare facilities, the general population, and more recently among animals. This worsens the prevalence of MRSA infections and poses a difficulty for infection control centers that mainly concentrate on healthcare-associated infections. Furthermore, although resistance to anti-MRSA agents usually occurs through bacterial mutation, there have been reports of transfer of resistance to antibiotics such as linezolid and glycopeptides, which is a major concern [7].

Fortunately, the incidence of MRSA infections associated with nursing seems to be decreasing because aggressive preventive hygiene measures in hospitals have had a positive effect, which confirms that infection control can limit the spread of MRSA [7]. On the other hand, over the past decade, the rate of community-acquired MRSA infections has increased rapidly among the general population. Although there is some evidence that these increases are trending downward, they do not follow the same downward trends observed for hospital-acquired MRSA infections [6].

*S. aureus* (including MRSA) is found on the skin and mucous membranes, and humans are the major reservoir for these microorganisms [38]. Approximately 50% of all people are thought to be colonized, and around 15% of the population carry *S. aureus* continuously in their nostrils [38]. Some populations tend to have higher rates of *S. aureus* colonization (up to 80%), such as healthcare workers, individuals who use needles regularly (i.e., patients with diabetes and intravenous drug users), hospitalized patients, and immunocompromised individuals. *S. aureus* can spread from person to person by direct contact or through contaminated objects [38,41].

*S. aureus* infections are regularly seen in primary care providers, internists, and infectious disease specialists. The primary objective of treatment is to ascertain the existence or non-existence of strains that are resistant to drugs. For most infections, it is recommended to limit the duration of antibiotic prescriptions to no more than 7–10 days [41]. The development of resistant bacteria is a consequence of the empirical prescribing of antibiotics [41]. Pharmacists should collaborate with the doctor to focus on antimicrobial therapy, while the nurse can oversee the progress to identify any need for adjustments in the treatment regimen if it proves to be ineffective. Such infections necessitate interprofessional collaboration in order to ensure correct treatment.

Furthermore, it is imperative that a multidisciplinary team of nurses and physicians provide the patient with comprehensive knowledge on hand hygiene to effectively mitigate the spread of illness to others. The treatment of *S. aureus* infections depends largely on the type of infection as well as the presence or absence of drug-resistant strains [41]. In general, penicillin remains the drug of choice for methicillin-susceptible *S. aureus* (MSSA) strains and vancomycin for MRSA strains [38]. Because many MRSA strains are resistant to multiple antibiotics, MRSA infections are increasingly recognized as serious pathogens in both hospital and community settings [38].

A recent study has shown the lethal effect of supernatant isolated from *S. aureus* under the effect of ciprofloxacin on MRSA strains [42]. The study involved the examination of 83 strains of *S. aureus* obtained from hospitals, and the investigation of the minimum inhibitory concentration (MIC) and minimum bactericidal concentration (MBC) of MRSA in the presence of ciprofloxacin. The existence of the *mecA* gene in the strains was confirmed through genotyping and phenotyping using PCR testing, revealing that all 83 samples harbored *mecA* genes, indicative of MRSA strains. The average MIC of ciprofloxacin and supernatant for various strains of MRSA were 0.032 mg/ml and 0.02 mg/ml, respectively [42]. Similarly, the average MBC of ciprofloxacin and supernatant for different strains of MRSA were 0.064 mg/ml and 0.04 mg/ml, respectively [42]. The impact of ciprofloxacin and supernatant on the mortality of stressed bacteria was verified, revealing the activation of genes associated with programmed cell death in many MRSA samples following bacterial stress induced by the antibiotic ciprofloxacin. Comparison of the MIC and MBC values for MRSA strains when exposed to ciprofloxacin and the liquid portion of the culture yielded similar outcomes. This suggests that the protein released by cultivated staphylococci has a lethal effect on the bacteria when combined with small quantities of ciprofloxacin [42].

### Haemophilus influenzae

Until about 1990, *Haemophilus influenzae* (*H. influenzae*) type b (Hib) was the leading cause of bacterial meningitis in children <5 years of age in the United States, accounting for 8,000–10,000 cases per year [43]. In addition, pyogenic arthritis, pneumonia, pericarditis, and facial cellulitis were all significantly attributed to Hib in young children, and it was also the main cause of epiglottitis [44]. With a peak incidence at 6–7 months of age, approximately 1 in 200 US children suffer from invasive (bacteremic) illness caused by this microorganism before turning 5 years old. Since 1990, the incidence of invasive Hib disease in the United States has decreased by more than 99%, to only 0.8 cases per 100,000 children under 5 years of age, primarily involving children who are not immunized or infants too young for vaccination [44–46]. Globally, Hib deaths decreased by 90% between 2000 and 2015 [47].

Although invasive Hib disease has been successfully reduced in the United States and other developed nations, Hib is still a frequent pathogen in countries in which a significant portion of the population lacks access to routine vaccinations, and continues to be the primary cause of bacterial meningitis and the second cause of bacterial pneumonia in these regions [48]. Roughly 900,000 cases of invasive Hib illness were recorded worldwide in 2008; meningitis and pneumonia accounted for the majority of the 199,000 fatalities [49]. This reflects a large decrease from approximately 371,000 deaths in 2000 [49].

Nontypeable strains of *H. influenzae* are a common cause of localized respiratory tract infections in children and adults, being the primary cause of purulent conjunctivitis, acute otitis media (AOM), otitis media with effusion, and sinusitis in children [50,51]. They are also a prevalent cause of pneumonia and a significant contributor to death in children residing in impoverished nations [50]. Nontypeable *H. influenzae* is also an occasional cause of serious invasive diseases such as septicemia, meningitis, and pyogenic arthritis, especially in neonates, pregnant women, and immunocompromised individuals [52,53]. Following the effective implementation of Hib vaccinations, nontypeable strains of *H. influenzae* currently account for the majority of invasive *H. influenzae* cases in the United States across all age categories. Between 2009 and 2015, the yearly occurrence of nontypeable *H. influenzae* invasive illness was approximately seven instances per 100,000 children aged under 5 years. In Europe, approximately 78% of all reported cases of *H. influenzae* were caused by nontypeable strains between 2007 and 2014 [54].

When considering the treatment of invasive *H. influenzae* infections, it is noteworthy that resistance to ampicillin is common among isolates, with a prevalence of >40% in some communities [55,56]. Consequently, ampicillin should not be used alone as empiric therapy for invasive disease. Resistance to ampicillin is usually related to beta-lactamase production, but is occasionally caused by reduced affinity of certain PBPs (especially PBP3) [57]. Ceftriaxone is the therapy of choice for meningitis caused by *H. influenzae* because of its potent activity against the bacterium (including beta-lactamase-producing isolates and isolates with an altered PBP) and because ceftriaxone reaches high levels in the cerebrospinal fluid (MIC_90_ ≤ 0.03, with rare isolates having an MIC of 0.25) [58].

Oral medications with efficacy against the strain responsible for otitis media, community-acquired pneumonia, and sinusitis can be used for treatment. Antibiotics that provide a MIC_90_ include cephalexin, cefaclor, cefuroxime, cefixime, amoxicillin-clavulanate, trimethoprim-sulfamethoxazole, clarithromycin, and azithromycin [58].

### Moraxella catarrhalis

*Moraxella catarrhalis* (*M. catarrhalis*) is responsible for up to 30% of AOM cases [59–61], and it is the second most common cause of exacerbation of chronic obstructive pulmonary disease in adults (after *H. influenzae*), being responsible for 2–4 million episodes each year in the United States [62].

Almost all contemporary isolates of *M. catarrhalis* produce beta-lactamase [63]. The bacterium has the ability to create biofilms in laboratory settings and has been found in biofilms in the middle ear of individuals suffering from chronic otitis media. Biofilm production by *S. pneumoniae* and *M. catarrhalis* is considered to be important in the role of these pathogens in recurrent AOM and serous otitis media [64,65]. At the mucosal level of the respiratory tract, *M. catarrhalis* activates a pro-inflammatory response and can also inhibit the inflammatory cascade, leading to the persistent colonization of the mucosal surface [66].

Colonization rates in the upper respiratory tract vary by age, with the highest rates (28–100%) in the first year of life [67]. *M. catarrhalis* can persist for several months, and earlier colonization is associated with higher risks of AOM and relapse [68]. Seasonal peaks of colonization and disease (winter and spring) are similar to several viral respiratory pathogens [69,70].

Between 5–32% of older adults with chronic obstructive pulmonary disease may have, at any time, *M. catarrhalis* colonizing the respiratory tract, with an average carrier duration of 30–40 days [71].

The most frequent diseases caused by *M. catarrhalis* are pneumonia, bronchitis, sinusitis, and AOM [72]. The majority of AOM cases are self-limited [72]. Compared with AOM caused by other pathogens, AOM caused by *M. catarrhalis* is more often a mixed infection and is less often associated with spontaneous perforation and mastoiditis [72]. The bacterium rarely causes suppurative complications of AOM, such as osteomyelitis, meningitis, or brain abscess. In recent years, *M. catarrhalis* has become a more common etiology of AOM, as cases of AOM with *S. pneumoniae* have decreased in frequency because of the routine use of pneumococcal conjugate vaccines (PCV7, PCV13) [61,73].

*M. catarrhalis* is almost uniformly resistant to penicillin, ampicillin, and amoxicillin owing to the production of beta-lactamases [74–77]. The drug of choice in the treatment of *M. catarrhalis* infections is amoxicillin-clavulanate. Second- or third-generation cephalosporins are alternative therapeutic agents. Strains with resistance to macrolides, tetracycline, trimethoprim-sulfamethoxazole, and quinolones have been reported [75,78].

The importance of *M. catarrhalis* as a respiratory pathogen in the post-PCV13 era and its increasing resistance to antimicrobial agents encourage consideration of a vaccine [79,80]. A combined nontypeable *H. influenzae* and *M. catarrhalis* vaccine candidate using the surface protein UspA2 demonstrated acceptable safety and immunogenicity in a phase 1 study in older adults [81].

### Vancomycin-resistant enterococci

The presence of VRE poses a significant treatment challenge. Enterococci are responsible for a wide range of diseases, especially among patients in hospitals or other healthcare institutions, including septicemia of various causes (surgery, urinary tract infections, etc.) [6,40]. VRE infections, often caused by *Enterococcus faecium* (*E. faecium*) and less often by *Enterococcus faecalis* (*E. faecalis*), have a lower worldwide prevalence and epidemiological impact than MRSA, except for the United States and some European countries [7].

The proportion of infections that are resistant to vancomycin depends on the species [6]. Overall, 30% of hospital-acquired enterococcal infections are resistant to vancomycin, resulting in 1,300 deaths per year [6]. The presence of *vanA* and *vanB* genes, responsible for vancomycin resistance, poses a significant risk, with some studies indicating the potential for gene transfer from enterococci to other bacteria, including *S. aureus* [7]. Antibiotic options for the treatment of VRE infections are limited [7]. Antibiotics used against VRE include linezolid and quinupristin–dalfopristin, whereas the role of daptomycin and tigecycline is still under investigation. Unfortunately, VRE remains a major threat. Consequently, there is tremendous interest in the development of new antibiotics that could have bactericidal action against VRE, such as oritavancin [7].

### Linezolid-resistant enterococci

The US Food and Drug Administration (FDA) approved linezolid as the first oxazolidinone antibiotic for use in clinical settings in 2000. Oxazolidinones, which are now used in hospital settings, have been regarded as a novel class of antibiotics for the last 40 years [82]. They are very effective against Gram-positive bacteria, including VRE and MRSA, and act through the suppression of protein synthesis by interacting with domain V of the 23S ribosomal RNA (rRNA) [83].

Linezolid is typically used to treat severe infections caused by Gram-positive bacteria that are resistant to many drugs. Currently, linezolid is regarded as a last-resort antibiotic [84]. However, for VRE infections, linezolid is the recommended course of action. Although highly transmissible VRE outbreaks were considered the source of linezolid resistance, it was shown that linezolid therapy in individual patients may also lead to the development of resistance in non-outbreak scenarios [84]. The possibility of VRE epidemics evolving into linezolid/vancomycin-resistant enterococci outbreaks is a significant worry, emphasizing the necessity of genetic surveillance as well as hospital outbreak management and monitoring [84].

Oxazolidinones inhibit the synthesis of bacterial ribosomal proteins and prevent the assembly of the initiation complex [85]. It was believed that resistance would not develop easily, as bacterial species frequently have several copies of the 23S rRNA gene (four alleles in *E. faecalis*, five–six alleles in *E. faecium*) and this would need changes in multiple 23S rRNA copies [86].

*Enterococcus* strains that are resistant to linezolid have become more common in recent years [87], and the most common causes include mutations in ribosomal proteins L3, L4, and L22, as well as domain V of the 23S rRNA [87,88].

The plasmid-mediated methyltransferase-encoding gene *cfr* was the first known transferable linezolid resistance gene [89]. The phenotype mediated by this gene confers resistance to lincosamides, phenicols, oxazolidinones, streptogramin A, and pleuromutilin [89]. Furthermore, it has been shown that the *cfr* gene is transferred between different bacterial species and genera [90,91]. In reference to enterococci, it was initially revealed that the *cfr* gene was present in animal-origin *E. faecalis*. Several more conjugative plasmids encoding this gene were discovered during additional research on enterococci [92]. In addition, reports of *E. faecium* isolates carrying the *cfr* gene are growing [93]. Since the *cfr* gene was discovered in a bovine *Staphylococcus* isolate in 2000, *Enterococcus* isolates from people and animals, including pigs, cattle, horses, and poultry, have also been shown to have this gene [94].

The ATP-binding cassette (ABC)-F protein encoded by the new transferable oxazolidone-resistance gene (*optrAIt*) in *Enterococcus* spp. was discovered in 2015. This protein confers cross-resistance to oxazolidinones and phenicols while mediating resistance through target protection [95–97]. The *optrAIt* gene is transferable and may be found on plasmids. It confers resistance to streptogramin B, aminoglycosides, macrolides, lincosamides, and phenicols, among other antibiotics. The *optrA* gene was initially identified in human-origin *E. faecalis*, and further investigations have revealed its existence in isolates of *E. faecium* [98–102]. It is more common in enterococci isolated from animals than from humans, according to monitoring studies [103]. The presence of the *optrA* gene was demonstrated in both *E. faecalis* strains isolated from veal meat (2015) and *E. faecium* strains isolated from turkey meat (2012) [104].

Recently, MRSA and enterococci were revealed to harbor *poxtA*, a gene that confers resistance to oxazolidinones [105]. It has been suggested that animal husbandry may be connected to *poxtA* [106]. In 2022, Zarzecka *et al*. reported that 28 strains exhibited phenotypic resistance to linezolid. Two strains (7.1%) were recognised as *E. faecium*, one strain (3.6%) was identified as *E. hirae*, and the majority (89.3%) belonged to the *E. faecalis* strain [82]. In total, 96.4% of the linezolid-resistant isolates were resistant to antibiotics from three or more classes, primarily ansamycins, tetracyclines, and macrolides, with linezolid MICs of 8–32 µg/ml [82]. In eight strains (28.6%), linezolid resistance was caused by the point mutation G2576T in domain V of the 23S rRNA. The *poxtA* gene was found in 64% of *E. faecalis* strains, whereas the *cfr* gene was found in 12% of *E. faecalis* strains and 50% of *E. faecium* strains [82]. The number of linezolid-resistant enterococci that have been identified since the drug was first used in clinical settings is continually rising [107,108]. They have been more prevalent in clinical isolates for a number of years, and lately, they have also been found in food [109–111].

There were fewer isolates of enterococci resistant to linezolid, according to an investigation of antibiotic resistance in foods derived from plants [112], suggesting that selection pressure caused by the use of antibiotics in animal husbandry may be the cause of resistance to linezolid [112].

The most prevalent causes of linezolid resistance are thought to be point mutations in the V domain of 23S rRNA and genes encoding ribosomal proteins L22 (*rplV*), L3 (*rplC*), and L4 (*rplD*) [113].

Zarzecka *et al*. have shown that the primary gene encoding for linezolid resistance is *poxtA* [82]. This gene was found in enterococci strains obtained from food-producing animals and animal-derived foods, and it has been demonstrated that eating certain foods can lead to human contraction of these bacteria [114]. According to Antonelli *et al*., strains with the *poxtA* gene may spread if oxazolidinones are used excessively in animals raised for food [105].

The available information demonstrates the significance of genotypic characterization of vancomycin and linezolid resistance, which may one day inform treatment decisions. Vancomycin can be used to treat isolates that are sensitive to both linezolid and vancomycin. Linezolid may be used as a monotherapy to treat VRE isolates. In rare cases, linezolid and daptomycin are given together [84]. Nevertheless, oral therapy is not available for linezolid/vancomycin-resistant enterococci isolates, necessitating intravenous antibiotic delivery [84]. Long-term usage of this antibiotic may lead to mutations that decrease resistance to linezolid, according to Smith *et al*. [115].

### Antibiotic-resistant *Mycobacterium tuberculosis*

The drug resistance of *Mycobacterium tuberculosis* (*M. tuberculosis*) poses a significant global problem. The WHO reported that in 2012, 170,000 people died from drug-resistant tuberculosis (TB) [9,41]. *M. tuberculosis* is most commonly spread by aerosols. The infections caused by this bacterium can occur anywhere in the body, but most often they are localized in the lungs [6].

The major factors driving TB drug resistance are incomplete, incorrect, or unavailable treatment, as well as the lack of new drugs [6]. Typically, TB infections can be treated and cured with first-line medications such as isoniazid or rifampicin. However, there are instances when *M. tuberculosis* may develop resistance to one or more of these drugs. The management of drug-resistant TB is intricate, necessitating extended treatment durations and the use of costly medications that frequently induce adverse reactions. Extensively drug-resistant TB (XDR-TB) is a form of TB that is resistant to the majority of drugs, including isoniazid, rifampicin, fluoroquinolones, and the three second-line injectable drugs (amikacin, kanamycin, and capreomycin). As a result, there are limited treatment options for patients with XDR-TB, and the effectiveness of available drugs is significantly reduced [6]. Although drug-resistant TB and XDR-TB infections represent a growing threat worldwide, including Romania, in some areas, such as the United States, they are uncommon owing to effective prevention measures [6].

### MDR *P. aeruginosa*

*P. aeruginosa* is a common cause of nosocomial infections, including pneumonia, urinary tract infections, post-surgical infections, and septicemia [6]. Approximately 400 deaths per year are attributed to these infections in the United States [6]. Unfortunately, some strains of MDR *P. aeruginosa* have been shown to be resistant to almost all antibiotics, including aminoglycosides, cephalosporins, fluoroquinolones, and carbapenems [6].

*P. aeruginosa* infections that have limited treatment choices are frequently observed in ICUs and long-term acute care hospitals. This is likely attributed to the excessive use of antimicrobial drugs, which facilitates the emergence and dominance of this bacterium [116]. The following are novel treatment options for MDR *P. aeruginosa* infections.

Ceftolozane–tazobactam is a fifth-generation expanded-spectrum cephalosporin paired with a widely recognised beta-lactamase inhibitor. This combination has heightened efficacy against *P. aeruginosa*, encompassing both MDR and XDR strains, owing to its ability to inhibit crucial PBPs. In addition, it demonstrated significant potency, primarily against Enterobacterales, including ESBL strains [117]. However, it is ineffective against *P. aeruginosa* strains that produce carbapenemase, which reduces the available treatment choices for carbapenem-resistant *P. aeruginosa*. Specifically, the presence of metallo-beta-lactamases (MBL) has been associated with the identification of *P. aeruginosa* strains that are not susceptible to ceftolozane–tazobactam [118,119]. Ceftolozane–tazobactam has demonstrated limited efficacy against *P. aeruginosa* in biofilm form in laboratory studies [120]. Ceftolozane–tazobactam is a favorable choice for treating susceptible MDR/XDR *P. aeruginosa* infections. It is considered a primary treatment option for carbapenem-susceptible *P. aeruginosa* strains according to recent European guidelines [121]. In addition, it is recommended for severe infections in the ICU and complex clinical situations, as demonstrated in real-life studies [122,123].

Ceftazidime–avibactam is a unique pairing of a widely used third-generation cephalosporin, recognised for its effectiveness against *Pseudomonas* bacteria, with a newly developed beta-lactamase inhibitor that does not belong to the beta-lactam class of antibiotics. This novel chemical exerts its effects by binding to PBPs found in the cell walls of Gram-negative aerobic pathogens and *P. aeruginosa*, including MDR or XDR strains [124,125]. Real-world observations regarding the treatment of MDR *P. aeruginosa* have shown promising levels of effectiveness. Firstly, in a group of patients with complex medical conditions and severe MDR Gram-negative infections, 31% of which were caused by *P. aeruginosa*, particularly those resistant to carbapenem antibiotics [126]. Secondly, in a retrospective study involving patients with MDR/XDR *P. aeruginosa* infections (61 initial episodes), although not treated immediately [127], it was found to be a viable treatment option. Furthermore, a significant proportion (87.8%) of severe infections caused by MDR and XDR *P. aeruginosa* isolates, which were not resistant to carbapenem, were successfully treated in a group of patients with Gram-negative infections caused by MDR, non-carbapenem-resistant Enterobacterales (CRE). This cohort consisted of 33 out of 41 cases (80.5%) of *P. aeruginosa* infections [128].

Imipenem–cilastatin–relebactam is a novel antibiotic combination that includes imipenem, a carbapenem, and relebactam, a powerful non-beta-lactam bicyclic diazabicyclooctane beta-lactamase inhibitor. Relebactam is chemically similar to avibactam but has an extra piperidine ring [129]. In 2019, the FDA authorized imipenem–cilastatin–relebactam for the treatment of complicated urinary tract infections (cUTI), including pyelonephritis, and complicated intra-abdominal infections (cIAI) in adult patients [129]. In 2020, the European Medicines Agency also approved it for the treatment of infections caused by aerobic Gram-negative bacteria in individuals with limited treatment alternatives [130]. Data obtained from the Study for Monitoring Antimicrobial Resistance Trends (SMART) surveillance program revealed that relebactam enhanced the effectiveness of imipenem in 80.5% of imipenem-resistant *P. aeruginosa* isolates in the United States [131]. Specifically, imipenem–cilastatin–relebactam demonstrated preserved effectiveness against 82.2% of MDR *P. aeruginosa* isolates and 62.2% of XDR *P. aeruginosa* isolates [132]. In another study, the susceptibility of *P. aeruginosa* isolates from intra-abdominal infections and the urinary tract to imipenem–cilastatin–relebactam was found to be 96.7% and 96.4% respectively. In addition, it was observed that imipenem-nonsusceptible and MDR *P. aeruginosa* strains had a susceptibility rate of 85% and 87.3%, respectively [133]. These data align with those from a Canadian study, which showed that imipenem–cilastatin–relebactam had an in vitro activity of 70.8% against MDR *P. aeruginosa* isolates [134].

Meropenem–vaborbactam is an antimicrobial combination that consists of a widely used, powerful carbapenem and a new cyclic boronic acid beta-lactamase inhibitor. The latter has a strong affinity towards serine residues, allowing it to act as a competitive inhibitor by forming a covalent bond with the beta-lactamase without being broken down through hydrolysis [135]. The efficacy of meropenem–vaborbactam against *P. aeruginosa* strains was determined to be generally comparable to that of meropenem alone. A study conducted by Lapuebla *et al*. revealed that 79% of *P. aeruginosa* isolates were sensitive to meropenem, and that the addition of vaborbactam did not alter this rate [136]. The main reason for meropenem resistance in *P. aeruginosa* strains is predominantly caused by mutations in porin or increased activity of efflux pumps. These mechanisms are not counteracted by vaborbactam [137]. However, a separate study indicated that the inclusion of vaborbactam resulted in enhanced eradication of bacteria in a neutropenic mouse thigh infection model, with certain strains of *P. aeruginosa*. This effect was observed despite the fact that the MIC of both agents was the same in laboratory tests. These findings suggest that these strains may possess an inducible beta-lactamase that is effectively inhibited by vaborbactam [138].

A recent study examined the effectiveness of meropenem–vaborbactam in treating pneumonia caused by *P. aeruginosa* and Enterobacterales. The study analyzed data from 3,193 *P. aeruginosa* isolates and 4,790 Enterobacterales isolates collected in US hospitals between 2014 and 2018. The results showed that 89.5% of *P. aeruginosa* isolates were susceptible to meropenem–vaborbactam. Among these isolates, the susceptibility rates for MDR strains and XDR strains were 59.0% and 48.6%, respectively [139].

### Carbapenem-resistant Enterobacterales

CRE are a group of bacteria that have become resistant to all or nearly all available antibiotics, including carbapenems, which are usually reserved as a last-resort treatment against pathogens resistant to other drugs [6,9,40]. The enzyme New-Delhi metallo-beta-lactamase-1 (NDM-1) is present in certain Gram-negative Enterobacterales (especially *E. coli* and *K. pneumoniae*), conferring resistance to practically all beta-lactams, including carbapenems [40].

Carbapenems are structurally similar to penicillin and are effective against a wide range of bacteria [140]. In contrast to other beta-lactams, carbapenems possess a carbon atom instead of a sulfone group at the fourth position of the beta-lactam ring. This distinctive architecture significantly contributes to their resistance against beta-lactamases [141]. Carbapenems have limited ability to pass through the cell wall, but they are able to enter bacteria by using outer membrane proteins known as porins. Carbapenems exert their action by breaking down the cell wall through the beta-lactam ring, specifically targeting the PBPs. The mechanism of action involves the degradation of the glycan backbone in the cell wall by autolysis, leading to the destruction of the cell as a result of osmotic pressure [140–142].

CRE refers to Enterobacterales that exhibit resistance to at least one carbapenem antibiotic, as determined by their antibiotic susceptibility profile (phenotypic definition) [143]. Carbapenem resistance primarily occurs when bacteria undergo certain mechanisms, such as genotypic changes. These mechanisms include acquiring structural alterations in PBPs, exhibiting a decrease or loss of specific outer membrane porins that prevent carbapenems from reaching their target site, activating efflux pumps to eliminate antibiotics and regulate the intramembrane environment, and acquiring beta-lactamases and carbapenemases to break down or hydrolyze carbapenems and other beta-lactam antibiotics (e.g., penicillins and cephalosporins) [140–143].

In general, CRE can develop resistance through genetic changes in the *porin* gene (in non-carbapenemase-producing CRE) or by developing enzymes that can break down carbapenem antibiotics (in carbapenemase-producing CRE) [143]. The existence or manifestation of the gene encoding carbapenemase often confers carbapenem resistance, affecting around 30% of CRE cases. Therefore, carbapenemase-producing CRE is a smaller group that falls inside the larger category of all CRE [143,144]. Although many individuals colonized with CRE do not develop illnesses, they can nevertheless transmit the bacteria [143]. Hence, the Antibiotic Resistance Laboratory Network and CDC laboratories perform regular tests for carbapenemase-producing CRE to proactively prevent and manage their onset and dissemination [143].

Carbapenemases, which are enzymes that break down carbapenem antibiotics, have been categorized into three groups based on the Ambler classification: class A, B, and D beta-lactamases. This classification is based on their ability to hydrolyze and be inhibited by certain substances, using either serine or zinc as catalysts [140,142,145,146].

Class A enzymes, namely serine beta-lactamases, catalyze the hydrolysis of a wide range of beta-lactam antibiotics, such as carbapenems, cephalosporins, penicillin, and aztreonam [146]. The enzymes are classified as chromosomally encoded and plasmid-encoded variants [146]. Chromosomally encoded enzymes include non-metalloenzyme carbapenemase-A (NMC-A), *Serratia marcescens* enzyme (SME), imipenem hydrolyzing beta-lactamase (IMI-1), and *Serratia fonticola* carbapenemase-1 (SFC-1). Plasmid-encoded variants include *K. pneumoniae* carbapenemase (KPC), imipenem-hydrolyzing beta-lactamase (IMI), and Guiana extended spectrum (GES) [140,145]. Of these, the KPC type is the most widespread enzyme and is responsible for epidemics in several Asian, African, North American, and European countries [140,145].

Class B enzymes, sometimes referred to as MBL, use metal ions, typically zinc, as a cofactor to target the active site of the enzyme, namely the beta-lactam ring. There are a total of ten varieties of MBLs, the most significant ones being New Delhi MBL (NDM), Verona integron-encoded MBL (VIM), and imipenemase (IMP) [140,143,145,147]. These enzymes break down all existing beta-lactam antibiotics, with the exception of monobactams such as aztreonam [148].

Class D enzymes, specifically serine beta-lactamases, are a group of enzymes known as oxacillinase (OXA) or oxacillin-hydrolyzing enzymes. There are more than 200 enzymes in this group. OXA exhibits fast mutation and a broad range of action. The most common carbapenem-hydrolyzing enzymes are OXA-48 and OXA-181, which exist in more than 40 different variations [149]. The prevailing forms of OXA-48 and OXA-101 in *K. pneumoniae* have been observed in Turkey, the Middle East, North Africa, and Europe [140,149,150]. Nevertheless, it is important to acknowledge that bacteria that produce OXA enzymes frequently exhibit resistance at a low level as a result of feeble expression. This poses a danger for the detection of false positive results and limits the availability of appropriate treatment alternatives [150].

Aztreonam, a monobactam antibiotic, is effective against bacteria that produce class B and D carbapenemases, when used alone. However, these bacteria frequently harbor ESBL genes, which hydrolyze aztreonam and render it ineffective. As a result, aztreonam has limited therapeutic use when used alone [151,152]. A potential therapeutic option for MBLs is the combination of aztreonam and ceftazidime–avibactam, a new beta-lactam-beta-lactamase inhibitor. Interestingly, aztre-onam is inactive against bacteria that produce class A carbapenemases, including those that produce the widely distributed KPC carbapenemases [151].

While ceftazidime–avibactam by itself is ineffective against MBLs, it exhibits a strong in vitro syn-ergy with aztreonam to act against these isolates [153]. This is especially crucial given that despite its effectiveness against class B carbapenemases, aztreonam is frequently broken down by other beta-lactamases that co-occur with MBLs [154]. Consequently, a recent global assessment revealed that only 29.2% of MBLs were still susceptible to aztreonam monotherapy, but all MBL isolates were inhibited by the combination of aztreonam and avibactam [155]. Six out of ten patients had clinical success after 30 days in a clinical case series assessing this combination treatment for infec-tions caused by NDM-producing MBLs during an outbreak. This suggests that ceftazidime–avibactam plus aztreonam may be a useful clinical option for XDR Enterobacterales infections that contain both class B carbapenemases and ESBL enzymes [156]. Based on these observations, the Infectious Diseases Society of America recommends the use of advises ceftazidime–avibactam alone CRE infections that produce OXA-48 outside of the urinary tract, and in conjunction with aztreonam for CRE infections that produce NDM [157].

Combinations of beta-lactam and beta-lactamase inhibitors have been developed and licensed in recent years with the express purpose of targeting organisms that are resistant to several drugs, such as CRE. The first of these, avibactam, was created in 2011. It is a synthetic diazabicyclooctane non-beta-lactam that exhibits action against class A (KPC) [158,159] and class D (OXA-48-like) carbapenemases, but not MBLs [159–161]. It binds to serine beta-lactamases covalently and re-versibly. Several observational studies have demonstrated that ceftazidime–avibactam is more ef-fective than polymyxin antibiotics in treating CRE infections, including class A carbapenemases, with less toxicity and adverse effects [162–165]. In 2015, the European Medicines Agency and the FDA authorized ceftazidime–avibactam in combination with metronidazole for the treatment of cIAI and cUTI [166]. The approval was based on the results of the RECLAIM trials, which have shown that ceftazidime–avibactam was not inferior to meropenem in the treatment of cIAI [167], and those of the RECAPTURE trial, which demonstrated that doripenem was not inferior to ceftazidime–avibactam in the treatment of cUTI [168]. Following the REPROVE study, a phase 3 trial conducted in 23 countries that demonstrated the noninferiority of ceftazidime–avibactam com-pared to meropenem for the treatment of nosocomial pneumonia, approval has recently been broad-ened to encompass hospital-acquired and ventilator-associated pneumonia [169]. According to the microbiological investigation, at baseline, a ceftazidime-resistant organism was present in 13.5% of patients in the RECLAIM trials, 19.6% of patients in the RECAPTURE trial, and 28% of patients in the REPROVE study. The rate of MBL infection was only reported by the RECLAIM studies, and it was around 3% [167].

In vitro susceptibility to ceftazidime–avibactam for CRE has remained high in isolates from hospitalized patients worldwide, according to data collected during the INFORM global surveillance survey for antimicrobial resistance; of the 816 non-MBL CRE isolates collected between 2012 and 2014, only 19 (2.3%) were resistant and 97.7% were susceptible to ceftazidime–avibactam [170]. Testing conducted on isolates obtained between 2015 and 2017 revealed a comparably high susceptibility to ceftazidime–avibactam, at 99.8% [171].

Although ceftazidime–avibactam susceptibility rates are still high overall, some mutations that confer resistance have been observed, mostly in carriers of KPC-2 and KPC-3 enzymes. It has been demonstrated that sequence type-258 *K. pneumoniae* with KPC-3 is resistant to ceftazidime–avibactam because KPC-3 was transposed onto a different plasmid, which changed the porin channels OmpK35 and OmpK36 and increased the expression of efflux pumps [172–174]. It is note-worthy that mutations in blaKPC-3 that confer resistance to avibactam have been observed in patients receiving ceftazidime–avibactam therapy. These mutations involve single amino acid substitu-tions at D179Y/T243M, D179Y, and V240G, which alter the Ω-loop in KPC-3. Nevertheless, in certain isolates, these mutations restore susceptibility to meropenem [175]. More recently, a three-amino-acid insertion was shown to confer greater affinity to ceftazidime and reduce the activity of avibactam, leading to resistance [176]. This KPC-3 variation, known as KPC-50, was identified from a *K. pneumoniae* isolate in a Swedish patient [176].

Patients in healthcare settings are increasingly experiencing infections from CRE bacteria that are incurable or challenging to treat. Each year, in the United States, approximately 600 deaths result from infections caused by the two most common types of CRE, carbapenem-resistant *Klebsiella* species and carbapenem-resistant *E. coli* [6].

### ESBL-producing Enterobacterales

ESBL-producing Enterobacterales contain a broad-spectrum beta-lactamase enzyme that favors the emergence of resistance to a wide variety of penicillin and cephalosporin antibiotics [6,9]. In the United States, Enterobacterales that produce ESBLs are responsible for 1,700 fatalities and 26,000 nosocomial infections annually [6]. A carbapenem antibiotic is a viable therapeutic option given that a significant fraction of ESBL-producing Enterobacterales are resistant to beta-lactam antibiotics. However, these drugs should be used with caution, as they contribute to the development of resistance [6].

### MDR *Acinetobacter*

*Acinetobacter* is a Gram-negative bacterium that causes pneumonia or bacteremia, particularly in individuals who are severely ill and require mechanical ventilation. Certain strains of *Acinetobacter* have developed resistance to nearly all antibiotics, including carbapenems, which are commonly regarded as the final option for treatment. Approximately 12,000 healthcare-related *Acinetobacter* infections occur in the United States each year, and 63% of these are MDR (resistant to at least three different classes of antibiotics), causing 500 deaths per year [6].

### MDR *N. gonorrhoeae*

In recent years, drug-resistant forms of *N. gonorrhoeae* have begun to appear in the United States [8]. Gonorrhea is characterized by discharge and inflammation of the urethra, cervix, pharynx or rectum [6]. Although not normally fatal, gonorrhea spreads easily and can cause severe reproductive complications [9]. The CDC estimates that over 800,000 cases of gonorrhea occur annually, being the second most common infectious disease reported in the United States [6]. If *N. gonorrhoeae* antibiotic resistance continues to spread, it is estimated to cause 75,000 additional cases of pelvic inflammatory disease, 15,000 cases of epididymitis, and 222 additional HIV infections over a projected 10-year period [6].

*N. gonorrhoeae* resistant to cephalosporins is often resistant to other types of antibiotics, such as fluoroquinolones (e.g. ciprofloxacin, levofloxacin, moxifloxacin, ofloxacin), tetracyclines and penicillins as well [6,7], therefore infections caused by these bacteria will not be able to be cured with empirical treatment regimens [6]. To address this challenge, the CDC has updated its guideline regarding the first-line treatment, recommending ceftriaxone with azithromycin or doxycycline [9].

## CAUSES OF ANTIBIOTIC RESISTANCE

### Mass use

The overuse of antibiotics is clearly leading to an exponential increase in resistance [6,177]. Epidemiological studies have shown a direct relationship between the use of antibiotics and the emergence and spread of resistant strains [178]. Antibiotics eliminate drug-sensitive microorganisms, leaving resistant bacteria to proliferate as a result of natural selection [178]. Despite warnings, antibiotics continue to be overused globally [178]. For example, in many countries, antibiotics are unregulated and available without a prescription [178]. This lack of regulation makes antibiotics easily available and affordable, thereby promoting overuse [179]. The possibility to purchase these products online further facilitates their availability, even in countries with strict regulations [179].

### Improper use

The misuse of antibiotics can also lead to the development of drug-resistant bacteria [6]. Several studies have shown that the indication, drug choice, or duration of antibiotic therapy is incorrect in 30% to 50% of cases [6,180]. Some antibiotics, even if partially effective, should be used with caution, especially in individuals with liver dysfunction, including liver fibrosis and cirrhosis [181]. In addition, 30–60% of antibiotics administered in the ICU have been found to be unnecessary, inappropriate, or suboptimal [180]. The misuse of antibiotics has questionable therapeutic efficacy and exposes patients to potential complications of antibiotic therapy [182]. Antibiotic resistance may arise as a result of subinhibitory and subtherapeutic antibiotic concentrations, as they can induce genetic alterations such as mutagenesis and altered gene expression. Antibiotic-induced changes in gene expression can increase virulence, whereas increased mutagenesis promotes and spreads antibiotic resistance [183]. The use of antibiotics in low concentrations has been shown to help diversify strains such as *P. aeruginosa*. Subinhibitory concentrations of piperacillin and/or tazobactam were also shown to be responsible for extensive proteome changes in *B. fragilis* [183].

### Widely used in agriculture

Antibiotics are used as growth supplements and as a means of preventing infection in animals in both developed and developing regions of the world. Similarly to humans, treating animals with antibiotics can cause bacteria to develop resistance. Antibiotic-resistant bacteria seen in animals can be pathogenic to humans, are easily transmitted to humans through the food chain, and spread widely in ecosystems through animal feces. In humans, this can lead to complex, untreatable long-term infections [184,185]. Antimicrobial products used for hygiene or cleaning purposes may also contribute to this problem as they may limit the development of immunity to environmental antigens in children and adults [179,8]. As a result, the multifunctionality of the immune system may be compromised, potentially increasing morbidity and mortality from normally avirulent infections [179].

### Availability of a small number of new antibiotics

The development of new antibiotics, which were previously successful in addressing antibiotic-resistant bacteria, has significantly decelerated owing to technical obstacles, limited understanding, substantial challenges in countering bacterial pathophysiology (such as the complex cell wall of Gram-negative bacteria), and financial and regulatory obstacles. Nevertheless, the widespread distribution of new antibiotics nearly invariably leads to the emergence of resistance, often occurring within a relatively brief timeframe. In an attempt to prevent this development, healthcare specialists frequently restrict the use of latest generation antibiotics, recommending them for the most severe conditions, and continue to administer already well-known antimicrobial agents (often generic drugs) that have demonstrated similar efficacy, thus increasing the likelihood that older agents become ineffective owing to the development of bacterial resistance [184].

### Global drug resistance: why antibiotic resistance is important

The economic impact of antibiotic resistance is substantial, being estimated to cost $55 billion annually in the United States [186]. Furthermore, research has shown that an infection caused by ESBL-producing *E. coli* or *Klebsiella* can increase hospital costs by an average of $16,450 and increase the length of stay by an average of 9.7 days [187].

The exorbitant costs associated with antibiotic resistance are not limited to developed countries. Developing countries are disproportionately responsible for the emergence of new antibiotic resistance genes, which have both domestic and international implications [188]. For example, NDM-1 was initially detected in a strain of *K. pneumoniae* from a Swedish patient who had recently traveled to India [189]. MRSA infections were found to result in substantial increases in overall mortality rates, bacterial infection mortality rates, mortality rates attributable to ICU, occurrence of septic shock, and a two-fold increase in the likelihood of long-term care [190]. Consequently, the management of MRSA imposes a considerable financial burden on healthcare organizations, although there is currently insufficient evidence to perform a comprehensive assessment of the economic impact. For example, antibiotics effective against vancomycin-resistant *S. aureus*, such as linezolid, are very expensive in developing or underdeveloped countries (e.g., in Romania, ten tablets of linezolid 600 mg cost approximately €145), significantly contributing to the spread of resistant strains [191]. Additionally, there is a lack of studies examining the economic implications of changes in the epidemiology of MRSA, such as infections acquired from farm animals [190].

According to the European Commission, there are 33,000 deaths caused by antibiotic resistance in the European Union each year, representing approximately €1.5 billion per year in healthcare costs [192]. According to a 2019 report of the CDC on the threat of antibiotic resistance, there are over 2.8 million cases of antibiotic resistance in the United States annually, 35,000 of which result in deaths. The emergence of carbapenem-resistant bacteria is a serious concern because they are classified as ‘last resort’ antibiotics for treating MDR infections [193].

The CDC’s assessment of antibiotic-resistant bacterial infections includes seven different factors, such as the clinical and economic impact of infections, case incidence, and projected incidence over a 10-year period. In addition, transmissibility, availability of effective antibiotics, and barriers to prevention are considered [6]. Based on this assessment, the CDC classified the threat level of each bacterium as ‘urgent’, ‘severe’, or ‘concerning’ (Table 1). Bacteria labeled as ‘urgent’ or ‘severe’ require more stringent monitoring and prevention measures, whereas those classified as ‘concerning’ require less immediate attention [6].

**Table 1:** The CDC’s assessment of antibacterial resistance threats [6]

**Urgent**	Carbapenem-resistant Enterobacterales Drug-resistant *N. gonorrhoeae*
**Severe**	Multidrug-resistant *Acinetobacter* ESBL Enterobacterales Vancomycin-resistant enterococci Multidrug-resistant *P. aeruginosa* Methicillin-resistant *S. aureus* Drug-resistant *S. pneumonia* Drug-resistant *M. tuberculosis*
**Concerning**	Vancomycin-resistant *S. aureus* Erythromycin-resistant *S. pyogenes*

## CONCLUSIONS

Antibiotic resistance is a widespread issue, with bacteria having developed mechanisms to counteract the effectiveness of antibacterial products over many millennia. The rise of antibiotic resistance, coupled with a lack of novel medications, presents a challenging future for antibiotic treatment. Once again, the significance of administering antibiotics in clinical practice cannot be minimized. There is a need for more efficient global regulation of antibiotic usage, even in developed nations. Discontinuing the use of non-prescription antibiotics and providing clinicians with knowledge regarding antimicrobial resistance could additionally diminish the usage of antibiotics. In order to mitigate insufficient demand, it is imperative to increase worldwide public awareness. The use of antibiotics in agriculture should be restricted to the management of contaminated animals rather than promoting growth. Enhancing the monitoring of antibiotic use and resistance is crucial to facilitate the implementation of antibiotic stewardship. In order to match the rise in antibiotic resistance, substantial global interventions and expenditures are anticipated in the manufacture of new antibiotics, funded by both public and commercial sectors.
